# A longitudinal study of *Campylobacter *distribution in a turkey production chain

**DOI:** 10.1186/1751-0147-51-18

**Published:** 2009-04-07

**Authors:** Päivikki Perko-Mäkelä, Pauliina Isohanni, Marianne Katzav, Marianne Lund, Marja-Liisa Hänninen, Ulrike Lyhs

**Affiliations:** 1 Finnish Food Safety Authority Evira, Research Department, Production Animal Health, PO Box 198, FI-60101 Seinäjoki, Finland; 2 Ruralia Institute, Seinäjoki Unit, University of Helsinki, Kampusranta 9C, FI-60320 Seinäjoki, Finland; 3 National Veterinary Institute, Technical University of Denmark, Hangøvej 2, DK-8200 Århus N, Denmark; 4 Department of Food and Environmental Hygiene, Faculty of Veterinary Medicine, University of Helsinki PO Box 61, FI-00014 University of Helsinki, Finland

## Abstract

**Background:**

*Campylobacter *is the most common cause of bacterial enteritis worldwide. Handling and eating of contaminated poultry meat has considered as one of the risk factors for human campylobacteriosis.*Campylobacter *contamination can occur at all stages of a poultry production cycle. The objective of this study was to determine the occurrence of *Campylobacter *during a complete turkey production cycle which lasts for 1,5 years of time. For detection of *Campylobacter*, a conventional culture method was compared with a PCR method. *Campylobacter *isolates from different types of samples have been identified to the species level by a multiplex PCR assay.

**Methods:**

Samples (N = 456) were regularly collected from one turkey parent flock, the hatchery, six different commercial turkey farms and from 11 different stages at the slaughterhouse. For the detection of *Campylobacter*, a conventional culture and a PCR method were used. *Campylobacter *isolates (n = 143) were identified to species level by a multiplex PCR assay.

**Results:**

No *Campylobacter *were detected in either the samples from the turkey parent flock or from hatchery samples using the culture method. PCR detected *Campylobacter *DNA in five faecal samples and one fluff and eggshell sample. Six flocks out of 12 commercial turkey flocks where found negative at the farm level but only two were negative at the slaughterhouse.

**Conclusion:**

During the brooding period *Campylobacter *might have contact with the birds without spreading of the contamination within the flock. Contamination of working surfaces and equipment during slaughter of a *Campylobacter *positive turkey flock can persist and lead to possible contamination of negative flocks even after the end of the day's cleaning and desinfection. Reduction of contamination at farm by a high level of biosecurity control and hygiene may be one of the most efficient ways to reduce the amount of contaminated poultry meat in Finland. Due to the low numbers of *Campylobacter *in the Finnish turkey production chain, enrichment PCR seems to be the optimal detection method here.

## Background

*Campylobacter *is the most common cause of bacterial enteritis worldwide. Commonly recognized risk factors are drinking surface water or water from private wells, swimming in natural waters, and drinking unpasteurised milk [1-5]. However, meat and especially the handling and consumption of undercooked poultry meat are considered as main risk factors for human campylobacteriosis [6-8].

*Campylobacter *contamination can occur at all stages of a poultry production cycle. In studies concerning vertical transmission, *C. jejuni *has been found on both outer and inner egg shell surfaces [9,10] and in the reproductive tract of laying and broiler breeder hens [11,12]. Hiet et al. [13] have shown the presence of *Campylobacter *DNA in fluff and eggshell samples. In contrast, Petersen et al. [14] and Herman et al. [15] reported no findings of *Campylobacter *from different samples collected in the hatchery e.g. incubator content, swab samples from hatchery machinery and floors and yolk sacs of diseased or dead chicks. Despite these observations, there is no clear evidence that vertical transmission or horizontal hatchery transmission does occur [14,16].

Many studies have provided strong evidence that the farm environment serves as a reservoir for the *Campylobacter *colonising poultry flocks. Dogs and other farm animals, wild birds, flies and untreated water may play a role in transmission of *Campylobacter *[17-21]. The prevalence of *Campylobacter *in broiler flocks varies in the different areas. Nordic countries like Finland, Sweden, Norway and Iceland have reported relatively low prevalences of 2,9%, 27%, 18% and 27,5% respectively [22-24]. In contrast, studies from other countries showed much higher occurrences of *Campylobacter *at the farm level, for example, 87.5% in the USA [25] and 42.7% in France [26]. Limited work has been carried out on investigating the prevalence of *Campylobacter *in the turkey production chain. Cox et al. [27] showed positive findings of 77% in male and 80% in female turkeys at 15 weeks of age. Other studies reported 48% and more than 80% of positive turkey flocks at the time of slaughter [28,29].

In spite of current cleaning and disinfection procedures, transport crates may be contaminated with *Campylobacter*, which may in turn contaminate birds during transport from the farm to the slaughterhouse [30,31]. During the slaughter process, contamination of the poultry carcasses and the equipment with *Campylobacter *occurs during defeathering, evisceration and the chilling processes [25,32]. Air also is found as a potential source of contamination at the slaughterhouse [33]. Contamination of turkey carcasses with *Campylobacter *at slaughter has been reported with levels of between 35% and 91.7% [34-37].

The aim of this study was to determine the occurrence of *Campylobacter *during one total turkey production cycle of 1,5 years time period, starting from imported parents (day-old chicks) to slaughter. For detection of *Campylobacter *at all stages of the production chain, a conventional culture method was compared with a PCR method. *Campylobacter *isolates from different types of samples have been identified to species level by a multiplex PCR assay.

## Materials and methods

### Study population and turkey production cycle

Between April 2005 and October 2006, one total turkey production cycle was studied. One cycle was defined as follows: Day-old parent chicks are imported from the UK. They are kept in parent rearing farms for 28 weeks. Before they start laying, the turkeys are transported to brooding farms, where they stay for 24 weeks. All the eggs they lay at the brooding farm are hatched in one hatchery. Day-old turkey chicks are transported to commercial farms. Turkey females and males are reared in the same house but separated by various types of walls. Following the slaughter of the females at 13–15 weeks, the males are allowed to use the entire house. Males are slaughtered at an age of 17–18 weeks.

At the parent rearing farm, the flock size was 2,700 and at the brooding farms the flock size was 2,300. Hatchery capacity was 900,000 poults per year. The size of the commercial farms varied from 6,000 to 18,000 birds per cycle. The slaughterhouse slaughtered only turkeys and the capacity was 3,500–5,000 birds/day. Only one flock was slaughtered per day.

### Collection and transport of the samples

All samples were collected on each occasion within 2 h, placed in a cool box and transported immediately to the laboratory, where they were processed within 2–4 h. Processing varied depending on the type of samples. Table 1 presents types and numbers of samples taken during this study.

**Table 1:** Places of sampling, types and numbers of samples taken during one total turkey production cycle.

Place of sampling	Type of samples	Number of samples (n)
Farm		
- Parent rearing farm	Paper liners, swabs from faecal droppings	80
- Parent brooding farm	Swab samples from droppings	70
- Rearing farm	Swab samples from droppings	360
		
Hatchery	Eggshells and fluff	30
		
Slaughterhouse	Caecal samples	120
	Environmental samples (swabs, water, faecal material)	336
	Neck skin samples	120
	Meat samples	60

Total number of samples (N)		456

For transporting swab samples from the farms and the slaughterhouse, each swab was put into a tube containing 37 g l^-1 ^Brain Heart Infusion Broth (LabM, Lancashire, UK) with 5% calf blood and 0.5% agar (Scharlau-Chemie, Barcelona, Spain) and stored at 4°C. In the laboratory, the swabs were placed into tubes containing 3 ml physiological saline (0.85% NaCl, w/v) and left to stand for five to 10 min to suspend bacteria before further processing.

Sterile gauze swabs (10 ×10 cm) were used to collect samples from the surfaces at the slaughter and meat-cutting departments. Before use, they were pre-moistened in Bolton selective enrichment Broth (Oxoid CM0983, Hampshire, UK) without supplement, placed in a sterile jar and stored at 4°C.

At the slaughterhouse, all environmental, neck skin and caecal samples were collected during the slaughtering process. At the same time, swab samples were collected from the transportation crates after disinfection and from the rubber boots of the workers in the evisceration room. Gauze samples were taken from different surfaces of the evisceration and cutting room and from the floor of the chilling room. All meat samples and environmental samples from the meat-cutting department were taken on the day of processing.

Process water samples of one litre were collected during the slaughter of each flock concerned from the defeathering machine and the chilling tank, respectively, into sterile plastic bottles.

### Samples

#### Faecal samples from parent rearing, brooding and commercial farms

At the first time of sampling in the parent rearing farm, ten samples were taken from the chick transportation bed including paper liners and faecal droppings. Thereafter ten swab samples were collected from fresh faecal droppings once every month over a period of seven months. After transfer of the birds to the brooding farm, ten swab samples were taken from fresh faecal droppings once every month, over a period of seven months. One swab was put into one transport tube. For enrichment, five swabs were pooled together to create two subsamples.

One to two weeks prior to the slaughter of females and males, 20 swab samples were taken from fresh faecal droppings at six rearing farms. The farms were randomly coded A to F. Five swabs were pooled together to create four subsamples. For enrichment, these four samples were pooled together.

#### Hatchery samples

Ten samples containing eggshell and fluff were taken three times over a period of three weeks and collected into separate plastic bags. In the laboratory, 20 g of each sample were measured into 180 ml Bolton selective enrichment broth (Oxoid CM0983, Hampshire, UK) with selective supplement (Oxoid SR0183) and 5% lysed horse blood for enrichment. In addition, 1 g was put into 10 ml physiological saline (0.85% NaCl) and left to stand for 10 min.

#### Caecal samples at the slaughterhouse

Ten caeca were taken at the evisceration line during the slaughter of each flock in question. Five caeca at a time were placed into one transport container. In the laboratory, each caecum was opened aseptically and swab samples from each caecum were taken. Five swabs were pooled to create two subsamples.

#### Environmental samples at the slaughterhouse

A total of 336 environmental samples were collected, consisting of swab, water, and faecal samples. The various sampling methods are described below:

A total of 180 gauzes were pre-moistened in Bolton broth (without supplement) and the different surfaces were wiped vigorously for 30 s. Gauzes were placed into a jars with 50 ml Bolton broth, without supplement. In the laboratory, 50 ml Bolton Broth with supplement was added to jars and mixed. The water samples were filtered in the laboratory through 0.45 μm filters (Fennolab, Vantaa, Finland) and four to eight filters were placed into 15–20 ml Bolton Broth (with supplement). Twenty-four litres of water were collected.

Faecal material from the transport crates was collected into a plastic bag. In the laboratory, 5 g of the material were placed into 45 ml Bolton broth (with supplement). A total of 12 faecal material samples were collected. Swab samples were collected as described earlier from transport crates (after disinfection) and from rubber boots in the evisceration room. Five swabs were pooled to create one sample.

#### Neck skin and meat samples at the slaughterhouse

Ten samples of neck skin were collected during the slaughter of each flock concerned. Furthermore, five meat samples consisting of a variety of cuts were collected separately into plastic bags from the meat-cutting department. In the laboratory, 25 g of neck skin (2 pooled samples of five times 5 g each) or meat (five separate samples 5 g each) were aseptically transferred into a Stomacher^® ^400 bag (Seward BA6041, Worthing, UK) containing 225 ml Bolton broth (with supplement) and shaken manually for 3 min.

### Culture method for detection of *Campylobacter*

All samples were tested by both direct plating and enrichment culture. Direct plating and isolation after enrichment was done on modified charcoal cefoperazone deoxycholate agar plate (mCCDA) (Oxoid CM739) supplemented with SR 155 (Oxoid). Plates were incubated at 42 ± 1°C for 48 ± 4 h under microaerobic conditions (5% O_2_, 10% CO_2_, 85% N_2_), generated by CampyGen™ (Oxoid CN0035). For enrichment, Bolton selective enrichment broth (Oxoid CM0983) with selective supplement (Oxoid SR0183) and 5% lysed horse blood was used and incubated at 42 ± 1°C for 22 ± 2 h under microaerobic conditions generated by CampyGen™ (Oxoid). The same enrichment and plating procedure was used for all samples described above.

### PCR method for detection of *Campylobacter*

For PCR, aliquots of 1 ml saline or Bolton broth, respectively, were collected from all samples both directly and after enrichment and centrifuged at 13,000 rpm for 8 min at room temperature. The supernatant was removed carefully and the pellet frozen at -80°C. DNA isolation from the frozen pellet was carried out using a DNA isolation kit, MagneSil^® ^KF Genomic System (Promega MD1460, Madison, WI, USA), with a Dynal MPC^®^-S magnetic stand (Dynal Biotech, Oslo, Norway) as described in Katzav et al. [38]. The detection of *Campylobacter *spp. in the samples was based on amplification of the 16S rRNA gene [39] using a set of oligonucleotide primers: C412F 5'-GGA TGA CAC TTT TCG GAG C-3' and 16S rRNA-campR2 5'-GGC TTC ATG CTC TCG AGT T-3' as described by Linton et al 1996 and Lund et al. [40], respectively. The internal amplification control (IAC) was prepared by isolating genomic DNA from the bacterium *Yersinia ruckeri *which is the causative agent of enteric redmouth disease in salmonid fish species [41]. This bacterium is not found naturally in chickens. For detection of the internal control, the primers Yers F8 5'-CGA GGA GGA AGG GTT AAG TG-3' and Yers R10 5'-AAG GCA CCA AGG CAT CTC TG-3' slightly modified from Gibello et al. [41] and slightly modified were used. All the primers were synthesised by Oligomer Oy (Helsinki, Finland). The PCR conditions used in the present study are described by Lund et al. [40] with a few modifications. Briefly, the PCR amplification was performed in 50 μl volumes containing 5 μl of the DNA, 25 μl of a PCR master mix (Promega, Madison, WI, USA), 1 μl of a 25 mM MgCl_2 _solution, 0.5 μl of a 10 mg ml^-1 ^BSA solution (New England Biolabs, Ipswich, MA, USA), 20 pmol of each of the *Campylobacter *primers and 5 pmol of each of the internal control primers and 10 pg of genomic Yersinia ruckeri DNA primers. The PCR was performed in a Peltier Thermal Cycler (PTC-200; MJ Research Inc., Watertown, MA, USA) and the conditions were one cycle of 95°C for 2 minutes, 58°C for 1 minutes, 72°C for 1 minute, followed by 34 cycles of 95°C for 15 seconds, 58°C for 40 seconds and 72°C for 40 seconds. The last elongation step lasted 5 minutes. The PCR product was loaded onto a 2% agarose gel (1.35% SeaKem^® ^LE Agarose and 0.65% NuSieve^® ^GTG Agarose, Cambrex Bio Science, Rockland, ME, USA) containing 0.1 g ml^-1 ^ethidium bromide. A DNA molecular weight marker 100 bp low ladder (P1473, Sigma-Aldrich, Saint Louis, MO, USA) was included in each gel. The gel was photographed under UV light (Alpha DigiDoc, Alpha Innotech, San Leandro, CA, USA). The PCR reaction for each sample was performed twice and considered positive if the primer set gave a distinct band of the right size (857 bp). Samples with no internal control band were run again using a tenfold dilution of DNA.

For sequencing of bands visible on the gel, PCR fragments was purified from the gel using an Qiaquick PCR purification kit (Qiagen GmbH Hilden, Germany) and sent for sequencing at DNA technology (Århus, Denmark) using the same primers for sequencing as used for the PCR. The homology of the sequenced PCR fragments to other *Campylobacter *sequences was determined using BLAST Sequence alignments.

### Identification of *Campylobacter *spp. isolates

Up to three *Campylobacter*-like colonies from each positive sample from rearing farms and slaughterhouse were selected, subcultured on mCCDA agar without supplement and incubated as described above. Identification to genus level was performed according to the method of the National Committee of Food Analyses [42]. To test their ability to grow in air, the colonies were streaked out onto blood plates (CASO agar, Casein-Peptone Soymeal-Peptone, Merck, Darmstadt, Germany with 5% bovine blood) and incubated aerobically at 37°C for up to three days.

For identification to species level, a multiplex PCR assay and two sets of primers based on the method described by Vandamme et al. [43] were used. The isolates were cultured on mCCDA agar without supplement and mixed with 20 μl of water and kept for 10 min at 100°C. The first primer set was *C. coli *specific, COL1 (5'-AG GCA AGG GAG CCT TTA ATC-3') and COL2 (5'-TAT CCC TAT CTA CAA ATT CGC-3') and the second set *C. jejuni *specific, JUN3 (5'-CA TCT TCC CTA GTC AAG CCT-3') and JUN4 (5'-AAG ATA TGG CTC TAG CAA GAC 3'). All primers were synthesised by Oligomer Oy (Helsinki, Finland). PCR amplification was performed in 25 μl volumes containing 3 μl of template, 12.5 μl of a PCR master mix (Promega, Madison, WI, USA), 1.5 μl of water and 20 pmol of each primer. PCR was performed in a Peltier Thermal Cycler (PTC-200; MJ Research Inc., Watertown, MA, USA) and the conditions were according to Vandamme et al. [43]. A DNA molecular weight marker 100 bp low ladder (P1473, Sigma-Aldrich, Saint Louis, MO, USA) was included in each gel. The gel was photographed under UV light (Alpha DigiDoc, Alpha Innotech, San Leandro, CA, USA).

### Data management and calculations

For data management and calculations Microsoft^® ^Excel 97 SR 2 was used. The level of agreement according to precision was expressed as the kappa statistic, defined as the proportion of potential agreement beyond chance exhibited by two tests. Diagnostic specificity was calculated as: *d*/(*b *+ *d*) where *d *is the number of samples negative both by PCR and by culture and *b *is the number of samples positive by PCR, but negative by culture. The level of agreement between two tests was calculated as: (*a *+ *d*)/*n*, where *a *is the number of samples positive both by PCR and by culture, *d *is the number of samples negative by both methods and *n *is the total number of samples under examination [44,45].

## Results

None of the 150 samples from the turkey parent flock, collected during the rearing and brooding period, and of the 30 samples from the hatchery were *Campylobacter *positive either by direct culture or culture following enrichment. However, using the PCR method, five samples from the parent flock in the brooding farm and one sample from the hatchery were *Campylobacter *positive. The PCR products from these samples were sequenced and identified as *C. jejuni*.

Table 2 shows the number of positive faecal samples in the six commercial farms (A-F) studied by culture and PCR method. Three farms (A, C and E) were found to be colonised with *Campylobacter *prior to slaughter. At farms A and E, both females and males were found positive. From farm C, only samples from the females were found *Campylobacter *positive whereas the males were negative at the first sampling. After transport of the females from farm C to the slaughterhouse, the male flock also became colonised with *Campylobacter*. No *Campylobacter *were found in the three other farms (B, D and F) either by direct and enrichment culture or by PCR method.

**Table 2:** *Campylobacter *colonisation in Finnish turkey rearing farms one to two weeks prior to slaughter and comparison of the conventional culture and PCR method for the detection of *Campylobacter.*

Sampling month	Farm	Direct culture		Enrichment culture		PCR		PCR after enrichment	
		
		Female	Male	Female	Male	Female	Male	Female	Male
July	A1^1^, A2^2^	4/4^3^	3/4	1/1	1/1	3/4	2/4	ND^4^	ND
August	A2		3/4		1/1		3/4		1/1
August	B1, B2	0/4	0/4	0/1	0/1	0/4	0/4	ND	ND
August	B2		0/4		0/1		0/4		ND
August	C1, C2	4/4	0/4	1/1	0/1	4/4	0/4	ND	ND
September	C2		3/4		1/1		4/4		1/1
August	D1, D2	0/4	0/4	0/1	0/1	0/4	0/4	ND	ND
September	D2		0/4		0/1		0/4		0/1
August	E1, E2	1/4	1/4	1/1	1/1	1/4	2/4	ND	ND
September	E2		1/4		1/1		0/4		1/1
September	F1, F2	0/4	0/4	0/1	0/1	0/4	0/4	0/1	0/1
October	F2		0/4		0/1		0/4		0/1

^1^Number one after the capital indicates female turkeys.

^2^Number two after the capital indicates male turkeys.

^3^Number of positive/number examined.

^4^ND. Not determined.

Table 3 provides details of the percentage of *Campylobacter *in the flocks at slaughter and at meat cutting. At the slaughterhouse, *Campylobacter *was isolated from at least one sample in 10 out of the 12 flocks studied. However, from two female flocks of the farms B and D no *Campylobacter *was detected. The female flock of farm B was *Campylobacter *negative also by PCR method, but PCR was not performed after enrichment from the samples of this flock. Generally, the percentage of *Campylobacter *of the samples taken during the slaughter process was higher than of those taken during the cutting process. In contrast, the meat samples of the males from farm D were all positive for *Campylobacter*, while only 75% to 83% the slaughter samples were positive.

**Table 3:** Prevalence of *Campylobacter *in turkey flocks during slaughter and meat cutting detected by culture and/or PCR method.

Farm	Processing plant				Meat samples			
	No. of positive/no. examined (%)				No. of positive/no. examined (%)			
	Female		Male		Female		Male	
	
	Culture^1^	PCR^1^	Culture^1^	PCR^1^	Culture^1^	PCR^1^	Culture^1^	PCR^1^

A	9/11 (82)	7/11^2 ^(64)	10/12 (83)	11/12 (92)	2/5 (40)	0/5 (0)	1/5 (20)	1/5 (20)
								
B	0/12 (0)	0/12^2 ^(0)	6/12 (50)	1/12^2 ^(8)	0/5 (0)	0/5 (0)	0/5 (0)	1/5 (20)
								
C	12/12 (100)	12/12 (100)	10/12 (83)	10/12 (83)	4/5 (80)	2/5 (40)	3/5 (60)	2/5 (40)
								
D	0/12 (0)	3/12 (25)	9/12 (75)	10/12 (83)	0/5 (0)	0/5 (0)	5/5 (100)	5/5 (100)
								
E	5/12 (42)	6/12 (50)	10/12 (83)	10/12 (83)	0/5 (0)	2/5 (40)	2/5 (40)	3/5 (60)
								
F	2/12 (17)	3/12 (25)	1/12 (8)	4/12 (33)	0/5 (0)	0/5 (0)	0/5 (0)	0/5 (0)

^1^No. of samples tested positive by direct and/or enrichment method.

^2^PCR not performed after enrichment.

Table 4 shows the number of *Campylobacter *positive samples taken at the processing plan. When using enrichment culture for *Campylobacter *determination, the highest percentage of positive samples was found in the environmental samples from the evisceration room (75%). Also faecal material collected from the transport crates (67%), the chilling water (67%) and the neck skins (62.5%) had high isolation rates after enrichment. Following enrichment, higher percentages of positive samples were observed among neck skin samples (62.5%) than among the caecal samples (33%). Environmental samples from the chilling- and cutting room were all negative by direct culture and direct PCR. However, following enrichment, 50% and 42% of the same samples from the chilling room, and 56% and 56% from the cutting room were found positive for *Campylobacter *by culture and PCR, respectively. Also water samples from the defeathering machine, neck skin samples, swab samples from the rubber boots of the workers in the evisceration room and meat cutting samples showed a higher percentage of *Campylobacter *using PCR after enrichment (Table 4).

**Table 4:** Occurrence of *Campylobacter *in samples at different stages and the environment of the slaughter and meat cutting departments detected by culture and PCR method

	Direct Culture	Enrichment culture	PCR	PCR after enrichment
	No. of positive/no. examined (%)	No. of positive/no. examined (%)	No. of positive/no. examined (%)	No. of positive/no. examined (%)

Transportation crates	1/11* (9)	1/11* (9)	1/11* (9)	1/9* (11)
Faecal material from transportation crates	7/12 (58)	8/12 (67)	7/12 (58)	7/9 (78)
Water from defeathering machine	0/12 (0)	5/12 (42)	3/12 (25)	5/9 (56)
Caecal material	9/24 (37.5)	8/24 (33)	8/24 (33)	8/18 (44)
Neck skin	2/24 (8)	15/24 (62.5)	6/24 (25)	12/18 (67)
Environment (evisceration room)	6/12 (50)	9/12 (75)	7/12 (58)	9/9 (100)
Rubber Boots (evisceration room)	3/12 (25)	6/12 (50)	3/12 (25)	5/9 (56)
Chilling water	3/12 (25)	8/12 (67)	3/12 (25)	7/9 (78)
Environment (chilling room)	0/12 (0)	6/12 (50)	0/12 (0)	5/9 (56)
Environment (meat cutting room)	0/12 (0)	5/12 (42)	0/12 (0)	5/9 (56)
Meat samples	0/60 (0)	17/60 (28)	4/60 (7)	13/45 (29)

*Eleven samples after washing and disinfection.

A total of 143 *Campylobacter *isolates from samples taken from the commercial farms and the slaughterhouse were identified as *Campylobacter *spp. by PCR. When species identification was performed using the multiplex PCR method, 105 isolates were identified as *C. jejuni *and none as *C. coli*. Thirty-eight isolates were not identified as either *C. jejuni *or *C. coli *by the multiplex PCR method. Thirty-four of these isolates originated from different slaughterhouse samples from both female and male flocks from farm C.

The diagnostic specificity for the comparison of direct PCR to direct culture was 0.88 with a level of agreement of 0.88 and for the comparison of both methods by selective enrichment was 0.88 with a level of agreement of 0.92.

## Discussion

*Campylobacter *contamination may occur at all stages of a turkey production cycle. In the present study, *Campylobacter *DNA was detected by PCR from five faecal samples collected during the brooding period. It is likely that the brooding flock had been in contact with *Campylobacter *but the infection had not spread within the flock. Self-limitation of colonisation and detection of antibodies against *C. jejuni *without colonisation of the bacterium has previously been described [17].

Detection of *Campylobacter *DNA by PCR in one fluff and eggshell sample supports the findings of Hiett et al. [13]. The bacterium was not isolated either from the present brooding flocks or from the hatchery and it is not possible to determine whether it is alive or dead. Thus, no further conclusions can be made on vertical transmission based on the present study.

The risk for *Campylobacter *contamination is high when strict biosecurity barriers are loosened and a poultry flock may come in contact with the environment via people and equipment on the farm. The possibility of compromising biosecurity during partial depopulation or "thinning" has yielded conflicting data. Several authors have demonstrated that the catching team can introduce the bacterium into the house, and therefore, partial depopulation has been considered a risk factor for *Campylobacter *colonisation [46-48]. In contrast, it has also been demonstrated that it does not necessarily influence *Campylobacter *colonisation in the flock [49]. At Finnish turkey farms, the flocks are usually divided and females and males are reared in separate groups but in the same house. Females are slaughtered two to four weeks before the males. After the turkey females have been slaughtered, the males can use the area where the females have been. This area could be seen as a risk for contamination since the personnel catching the turkeys can break the hygiene barriers during collection of the female birds. In this study, three flocks were *Campylobacter *negative before slaughter of the females and remained negative when testing the males two to three weeks later. Hansson et al. [50] found no differences in the presence of *Campylobacter *in the environment between producers who frequently or rarely deliver *Campylobacter *positive slaughter batches. Thus, our results could be explained by good hygiene control of the catching equipment and personnel in the negative farms.

The slaughter process was found to be a risk factor for the *Campylobacter *contamination of turkey products. The number of *Campylobacter *positive samples within a flock at slaughter varied between 0 and 94% in this study. High variation in the turkey flocks at the slaughterhouse has also been demonstrated previously [35,37]. Since enrichment was needed to recover the bacteria, it seems that some processing steps like the scalding and defeathering process had an adverse effect on the bacteria. This study found more positive neck skin samples than caecal samples (Table 3). Neck skins are mentioned as good indicators of *Campylobacter *contamination at the slaughterhouse [32]. Hansson et al. [31] found more *Campylobacter *from neck skin samples than from cloacal samples and concluded that if cloacal samples were negative, the neck skin samples might have been contaminated from the slaughterhouse environment. This may also explain the results of the present study.

Evisceration is a critical stage where bacteria can be spread in poultry processing. This fact is confirmed by this study, showing samples from the evisceration room and rubber boots to be 50 to 100% *Campylobacter *positive. It has been shown that contamination at the slaughterhouse cannot be avoided when a *Campylobacter *positive poultry flock is processed [15]. Allen et al. [51] isolated *Campylobacter *at a slaughterhouse from aerosols, particles and droplets in the hanging, plucking and evisceration areas also during the processing of a *Campylobacter *negative flock. In this study, all slaughtered birds originated from the same flock and only one flock per day was slaughtered. Thus, cross-contamination from another, potentially positive, flock slaughtered earlier the same day was not possible. However, in this study there is also evidence that contamination at a slaughterhouse can withstand cleaning and disinfection. Flocks B2, D2, F1 and F2 were *Campylobacter *negative at the farm level, caecum culture-negative at slaughter, but tested positive during the slaughter process. Peyrat *et al*. [52] also recovered *C. jejuni *from the equipment surfaces after cleaning and disinfection in three out of four slaughterhouses visited. It is possible that *Campylobacter*, as well as other bacteria, persist on surfaces in poultry-processing facilities forming a biofilm [53-55]. Thus, the release of the bacterium from such biofilms may also contaminate products which touch the surface of the processing equipment.

In the slaughterhouse studied here, the turkey carcasses were chilled by placing them first in a water tank for five minutes before hanging them for 24 hours in a room at 2°C. More positive samples from the chilling water than from the chilling room environment were observed, suggesting the chilling water as being a source of carcass contamination. Extended air-chilling might lead to drying of the carcass surface and the environment of the chilling room resulting in a reduction of *Campylobacter *[51,56,57]. In this study, the occurrence of *Campylobacter *in the samples taken during the meat cutting process was lower than of those taken during the slaughter process. In the present slaughterhouse, the meat was cut the day after slaughter. It is known that certain subpopulations of *Campylobacter *are able to survive environmental stress like the scalding- and chilling process and remain in the final meat products [58]. However, the low rate of *Campylobacter *in the final meat products found in the present study (28%) is reflected by the low findings in poultry products at the Finnish retail level with reported numbers of 12% and 21%, respectively [38,59].

Of the 143 *Campylobacter *spp. isolates, 105 (73%) were identified as *C. jejuni*, none as *C. coli*, so 38 (26%) remained unidentified to the species level. It is known that the majority of the *Campylobacter *found in raw poultry are *C. jejuni *[37,57,60]. Takahashi et al. [61] found both *C. jejuni *and *C. coli *in farm samples, *C. jejuni *at all stages of the processing line. However, they did not find *C. coli *anymore after defeathering and speculated lower numbers of *C. coli *in poultry faeces to be the reason. Certain *C. jejuni *strains might be more stress-resistant and overgrow possible *C. coli *strains in the same samples [58].

As the high level of agreement between the different detection methods shows, there were no significant differences between the conventional culture and the PCR method in the samples analysed in this study. However, the need for enrichment in this study for the detection of *Campylobacter *at certain processing steps, also when performing PCR, might indicate low numbers of *Campylobacter *at the farm and slaughterhouse level. Thus, a combination of enrichment and PCR assay seems to be the optimal method for detection of *Campylobacter *in this situation.

## Conclusion

The presence of *Campylobacter *DNA from the brooding flock and hatchery sample shows that they have been in contact with *Campylobacter*, but for unknown reasons the contamination has not been spread. The present study also shows that during the processing of a *Campylobacter *positive turkey flock, working surfaces and equipment at the slaughterhouse can become contaminated, leading to possible contamination of negative flocks, even if slaughtered on following days. Persistence of *Campylobacter *on surfaces emphasises the need for efficient cleaning and disinfection of the processing facilities. However, the need for enrichment in this study for detection of *Campylobacter *at certain processing steps, also when performing PCR, might indicate low numbers of *Campylobacter *at the farm and the slaughterhouse level. Since complete elimination of thermophilic *Campylobacter *from the turkey production chain does not seem feasible, reduction of contamination at the farm level by a high level of biosecurity control and hygiene may be one of the most efficient ways to reduce the amount of contaminated poultry meat in Finland.

## Competing interests

The authors declare that they have no competing interests.

## Authors' contributions

PPM, UL, MK, PI and M-LH participated in the discussion on the study design. PPM, UL, MK and PI participated in the collection of samples, analysis and interpretation of the data. PPM, UL, MK and PI carried out the microbiological analyses of the samples. Analysis and interpretation of the PCR were carried out by PPM. Analysis and interpretation of the sequencing were carried out by ML. PPM and UL wrote the manuscript. All authors read and approved the final manuscript.
